# De Novo Myeloid Sarcoma of the Kidney: A Case Report and Literature Review of Clinical Features and Outcomes

**DOI:** 10.7759/cureus.95266

**Published:** 2025-10-23

**Authors:** Jie Xiong, Sherryn Sherryn, Pengfei Zhang, Lin Yang, Shenghan Wang, Bagus Baskoro, Bao Zhang

**Affiliations:** 1 Department of Urology, Aerospace Center Hospital, Beijing, CHN; 2 Department of Urology, Peking University Health Science Center, Beijing, CHN; 3 Department of Urology, Universitas Indonesia, Jakarta, IDN; 4 Department of Urology, Dr. Cipto Mangunkusumo National Central Public Hospital, Jakarta, IDN; 5 Department of Pathology, Aerospace Center Hospital, Beijing, CHN; 6 Department of Urology, Pasar Rebo Regional General Hospital, Jakarta, IDN

**Keywords:** case report, de novo myeloid sarcoma, myeloid sarcoma, surgery, uro-oncology

## Abstract

Myeloid sarcoma (MS) can manifest as a primary disease entity, known as de novo myeloid sarcoma, without the concurrent presence of acute or chronic myelocytic leukemia, myelodysplastic syndrome, or myeloproliferative neoplasm. It is crucial to approach suspected de novo myeloid sarcoma cases comprehensively to prevent misdiagnosis and ensure timely and accurate treatment.

We report a case of de novo myeloid sarcoma involving the kidney in a 38-year-old female patient with a chief complaint of a rapidly enlarging palpable mass in the upper right quadrant for over a month. The patient’s enhanced abdominal CT scan result was initially suspected to be renal cell carcinoma. Subsequently, we performed radical nephrectomy and post-operative pathology, immunohistochemistry, and cytogenetic analysis to confirm the diagnosis of de novo myeloid sarcoma. Histopathological analysis confirmed malignancy originating from the right kidney, accompanied by necrosis. The tumor cells displayed moderate size, evident pleomorphism, and off-centered granular cytoplasm. Extensive infiltration of the renal parenchyma was observed, involving the renal capsule, perirenal adipose tissue, and renal pelvis mucosa. CD117 displayed positive immunohistochemical staining.

There is currently a dearth of studies that comprehensively describe de novo myeloid sarcoma involving the urogenital tract. In light of this gap in the literature, the present study aims to provide a systematic literature review and present a case of de novo myeloid sarcoma involving the kidney in patients with normal bone marrow biopsy. Our objective is to elucidate the clinical presentation and prognosis of de novo myeloid sarcoma involving the urogenital tract, thereby contributing to the existing knowledge base, and facilitating evidence-based treatment decisions. The multifaceted nature of MS necessitates a multidisciplinary approach, including thorough diagnostic evaluation involving immunohistochemistry, cytochemistry, and cytogenetic analysis. Standardizing terminology and refining diagnostic and treatment algorithms through future prospective studies are vital steps toward enhancing clinical management and prognosis for patients with de novo myeloid sarcoma of the urogenital tract.

## Introduction

Myeloid sarcoma (MS) is a rare neoplastic disorder characterized by the presence of myeloid blasts, according to the World Health Organization (WHO) classification of hematolymphoid tumors [1]. MS can manifest as a primary disease entity, known as de novo MS, without the concurrent presence of acute or chronic myelocytic leukemia, myelodysplastic syndrome, or myeloproliferative neoplasm [2,3]. Alternatively, it can coincide with the manifestation of these hematological disorders. The tumor typically occurs outside the bone marrow, affecting various regions of the body, including the bone, skin, or lymph nodes, leading to effacement of tissue structure [1].

While the exact etiology of MS remains unknown, it has been hypothesized that it shares similarities with acute myeloid leukemia (AML). In patients with histopathological normal bone marrow or de novo MS, the presence of relevant gene mutations indicates the potential existence of clonal hematopoiesis or low-level clonal myeloid disease in the bone marrow [3-5]. The pathological characteristics of MS involve infiltration of affected organs by myeloblasts, which can efface the structural integrity, and in some cases, promyelocytes or neutrophils may also be present.

The prevalence of de novo MS without bone marrow involvement is rare, with a reported incidence of two per 1,000,000 in adult patients, as reported by Antic et al. in their study [6]. Furthermore, isolated MS involving the urogenital tract is exceptionally rare, with only limited case reports and case series indexed in Medline. The diagnosis of MS, particularly when involving the urogenital tract, poses significant challenges for urologists due to the rarity of the disease. It is crucial to approach suspected de novo MS cases comprehensively to prevent misdiagnosis and ensure timely and accurate treatment. In addition to biopsies, molecular and cytogenetic studies are recommended to aid in the diagnostic process [7].

Despite the increasing number of case reports and case series on de novo MS in other systems, there is currently a dearth of studies that comprehensively compare treatment options, particularly for MS involving the urogenital tract, the clinical knowledge and prognosis of de novo MS in this specific context are primarily derived from published case reports or case series. In light of this gap in the literature, the present study aims to provide a systematic literature review and present a case of de novo MS involving the kidney in patients with normal bone marrow biopsy. Our objective is to elucidate the clinical presentation and prognosis of de novo MS involving the urogenital tract, thereby contributing to the existing knowledge base, and facilitating evidence-based treatment decisions.

This report follows the CARE (CAse REport) guidelines that employ a case reporting checklist and addresses the rarity and diagnostic challenges associated with de novo MS involving the urogenital tract [8]. By conducting a systematic literature review and presenting a unique case, we strive to enhance the understanding of the clinical characteristics, diagnostic approaches, and treatment options for this rare neoplastic disorder.

## Case presentation

A 38-year-old female patient of Asian descent was referred to our institution with a primary complaint of a rapidly enlarging palpable mass in the upper right quadrant of her abdomen, which had been progressively growing for over a month. The patient also noted an unintentional weight loss of five kilograms during this period but did not report experiencing any other significant symptoms.

Before being admitted to our hospital, the patient had undergone both abdominal ultrasound and computed tomography (CT) scan at a local medical facility. Radiographic investigations unveiled a large, intricate mass within the right kidney characterized by a combination of cystic and solid components. Due to the suspicion of malignancy, the patient was subsequently referred to our facility for further diagnostic evaluation.

Upon physical examination, a painless mass was palpable in the right upper quadrant of the abdomen. The dimensions of the mass were approximately 10 cm in diameter, with ill-defined margins. The patient had an unremarkable medical history and denied any chronic illnesses or prior surgical interventions. The patient’s father had a diagnosis of thyroid papillary carcinoma, while the rest of her family history was unremarkable. Additionally, the patient's past medical record included a history of cervical disc herniation and radiculopathy spanning two months, which had been managed using acupuncture.

The patient denied any habitual alcohol consumption or nicotine use. Blood tests revealed that the patient has mild anemic condition, with a hemoglobin level of 102 g/L (reference range: 121-151 g/L). A complete blood count displayed no notable abnormalities, with the white blood cell count at 9.63×10^9^/L (reference range: 4.0-11.0×10^9^/L).

Upon admission to our care, the patient exhibited fever of uncertain etiology, suspected to have a malignant origin. The patient had an allergy to sulfonamides. Despite administration of prescribed antibiotics, empirical treatment using cephalosporin and levofloxacin proved ineffective.

**Figure 1:**
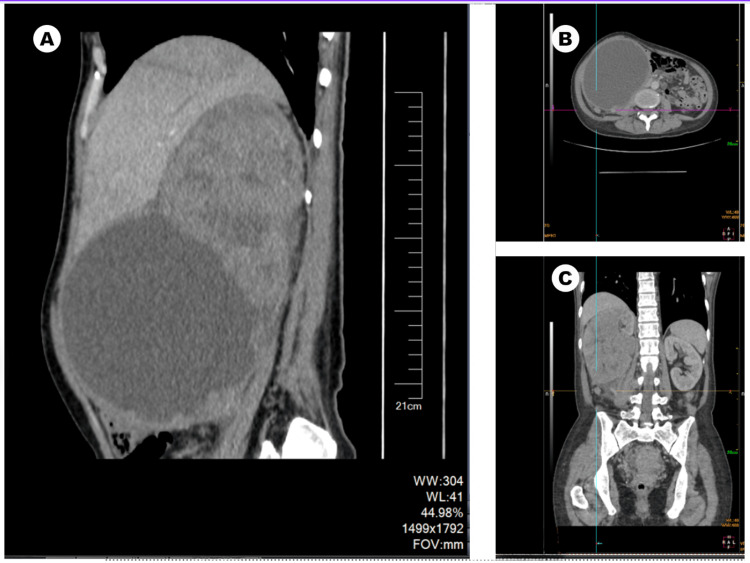
Abdominal CT scan with contrast of the patient. (A) Sagittal plane of the abdominal CT scan showing the mass; (B) transverse plane; (C) coronal plane.

Consequently, an abdominal CT scan with contrast was conducted to further investigate the case, which revealed a mass of mixed density, composed of both solid and cystic components as can be seen in Figure 1. The solid portion measured roughly 12.0×9.9×14.1 cm and exhibited enhancement values of 39 Hounsfield Units (HU), 46 HU, and 49 HU during different stages of the enhancement phase. The cystic component, measuring approximately 14.9×12.7×14.3 cm, displayed an enhancement value of 11 HU. The multiseptated cystic wall exhibited irregular nodular changes and uneven thickening. Adjacent organs displayed signs of compression, with the right renal vein and its branches exhibiting indistinctness, potentially suggesting their involvement. Multiple retroperitoneal lymph nodes were enlarged, with the largest measuring approximately 0.8 cm in diameter. The CT scan findings strongly indicated malignancy, leading to the decision to proceed with radical nephrectomy.

The surgical procedure was performed with the patient in a left lateral decubitus position. An initial 5 cm transverse incision was made to the right of the umbilicus. The tumor was found to be solid and firmly adhered in place. The surgeon decided to extend the incision from the umbilicus to the xiphoid process, forming a "7" shape, facilitating exploration beneath the tumor. Notably, the tumor exhibited adhesions with the ascending colon on its distal aspect, the duodenum on the medial aspect, the quadratus lumborum on the lateral aspect, and the liver on the proximal aspect. After achieving adequate exposure, careful dissection freed the ascending colon from the tumor's lower pole, and the duodenum was separated from the medial aspect. The right renal vein was identified, dissected, and separated, with some difficulty encountered during the separation of the posterior renal artery. Gentle lifting of the lower pole of the kidney facilitated the tumor's dissection from the posterior aspect, carried out using a harmonic scalpel. Additionally, right adrenalectomy was performed. Management of the renal hilum was executed using a laparoscopic stapler, leading to complete excision of the tumor with intact margins. Hemostasis was accomplished using a bipolar coagulator, followed by closure of the wound using interrupted sutures. Notably, the duodenum remained intact, and no bleeding was observed in the inferior vena cava. Minor defects were observed in the muscularis propria layer of the colon wall, which were subsequently sutured. Throughout the surgical procedure, effective hemostasis was notably challenging owing to extensive adhesions involving the tumor, surrounding organs, and structures. Moreover, the patient exhibited a mild state of anemia. Consequently, in the course of the operation, the estimated blood loss was 300mL, and the patient required an infusion of 10 units of blood and 800 ml of plasma.

**Figure 2:**
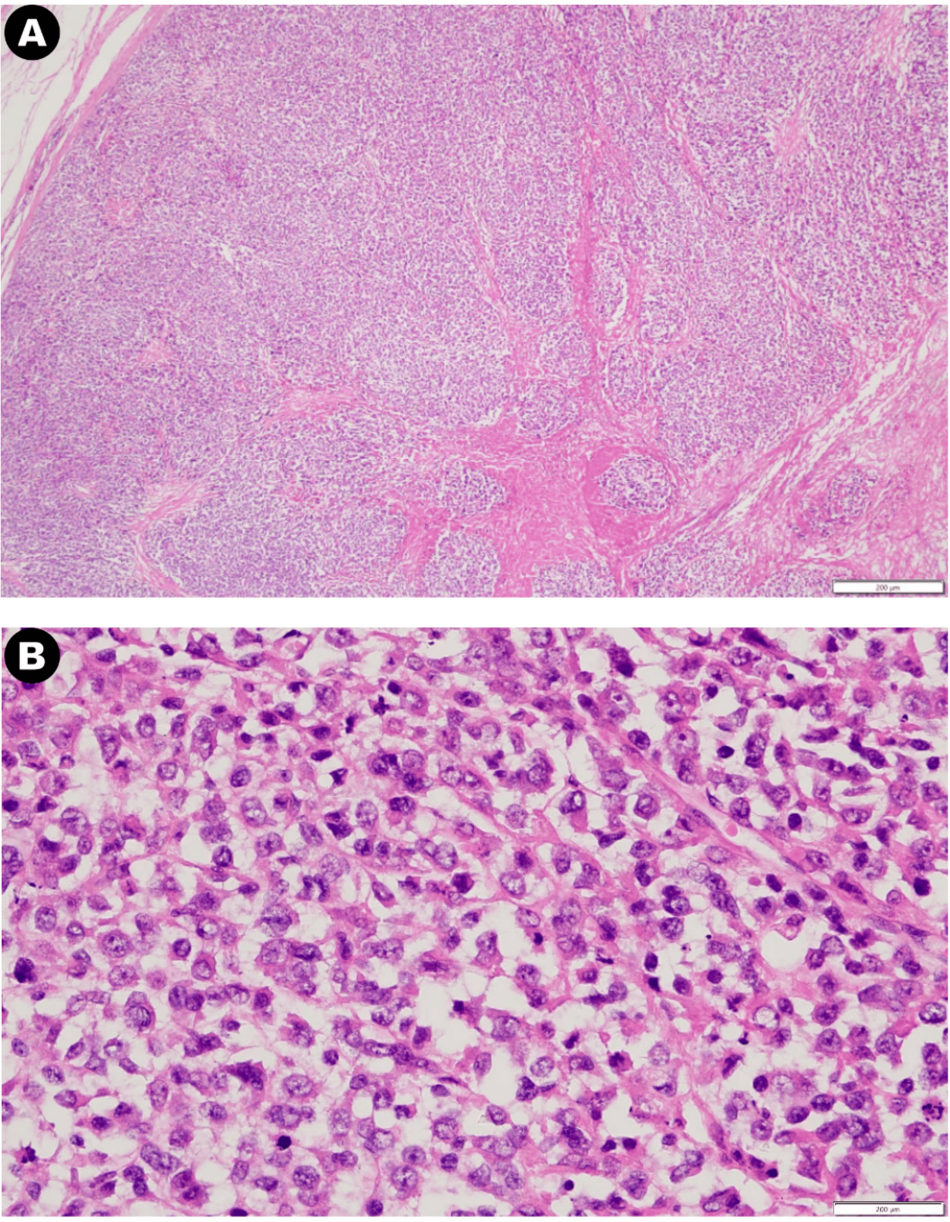
Histopathological slides of the resected abdominal mass specimen. (A) Histopathological slides of abdominal mass; (B) Moderate size tumor cells with evident pleomorphism, and off-centered granular cytoplasm.

Upon examination, the excised specimen measured approximately 16x12x10 cm, complete with an intact capsule. Upon dissecting the cystic area, the cystic fluid displayed a light-yellow hue. Sectioning the specimen revealed thickened cyst walls with a yellow appearance on the inner surface within the cystic region. The solid area exhibited a firm texture, with septa and a grayish-white coloration. Histopathological analysis can be seen in Figure 2, with confirmed malignancy originating from the right kidney, accompanied by necrosis. The tumor cells displayed moderate size, evident pleomorphism, and off-centered granular cytoplasm. Extensive infiltration of the renal parenchyma was observed, involving the renal capsule, perirenal adipose tissue, and renal pelvis mucosa.

Tumor involvement was noted in the peripheral tissue of the renal vein stump, while the ureteral stump exhibited no tumor infiltration. Additionally, the right para-aortic lymph nodes showed tumor involvement, and the adrenal gland was affected by tumor tissue. Based on the results of immunohistochemical staining, myeloid sarcoma was considered as a potential diagnosis. Immunohistochemistry results were as follows: CD3 (-), CD20 (-), CK (-), Vimentin (+), CD117 (+), MPO (-), MyoD1 (-), PAX-8 (-), CD10 (-), MUM1 (-), Bcl-6 (weakly positive), Syn (-), ALK (-), CD38 (-), CD138 (-), CD4 (-), CD8 (-), PAX5 (-), CD30 (-), GranzymeB (-), TIA-1 (-), EMA (-), Ki-67 (+ 90%), Lysozyme (-), and CD19 (-). Notably, among these markers, only CD117 and Ki-67 displayed positive immunohistochemical staining.

**Figure 3:**
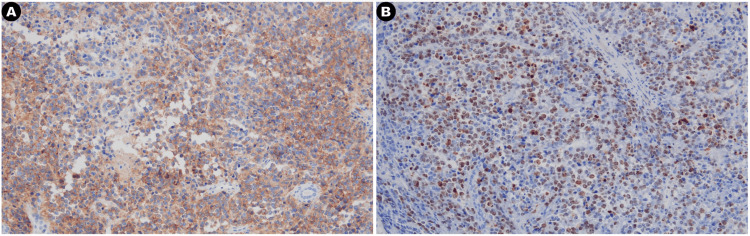
Immunohistochemistry slides of the resected abdominal mass specimen. (A) CD117; (B) Ki-67.

The patient recovered well from the surgery and consented to be presented in this case report. The patient was subsequently transferred to be managed by the healthcare professional team in the oncology department.

## Discussion

In this study, we presented a case of de novo myeloid sarcoma of the kidney and conducted an extensive systematic literature search with a specific focus on de novo MS cases affecting the urogenital tract. The objective was to comprehensively explore various facets of these cases, encompassing patient demographics, anatomical sites of occurrence, clinical presentations, laboratory findings, treatment responses, and prognostic outcomes. A meticulous search was performed on PubMed on May 2, 2023, without imposing any date restrictions.

There were challenges in finding all the relevant articles due to misnomers and multiple names used to diagnose MS prior to the unification of terms published in the WHO Classification of Tumours of Haematopoietic and Lymphoid Tissues [3,9]. This inconsistency in nomenclature has contributed to confusion in understanding the epidemiology and prevalence of the condition, often resulting in studies claiming to report the first instance of de novo MS involving the urogenital tract.

By employing controlled vocabulary and medical subject heading (MeSH) terms, we successfully identified 13 studies encompassing a total of 14 patients with a diagnosis of MS involving the urogenital tract without evidence of bone marrow involvement [10-22]. The range of publication dates extended from 1984 to 2021. Detailed summary of the included studies is presented in Table 1.

**Table 1:** Literature review of de novo MS involving the urogenital system.

Study Citation	Methodology (country; study design)	Participant Characteristic	Clinical Presentation (classification; site; clinical symptoms)
Al-Quran et al. (2006) [10]	USA; Case report	47, male	De novo MS; Bladder and epididymis; Hematuria, flank pain, space-occupying symptoms
Acar et al. (2013) [11]	Turkey; Case report	41, male	De novo MS; Ureter; Gross hematuria, flank pain, hydroureteronephrosis
Aki et al. (2002) [12]	Turkey; Case report	36, male	De novo MS; Bladder; Fatigue, pollakiuria, hematuria, suprapubic region tenderness
Chaitin et al. (1984) [13]	USA; Case series	29, female	De novo MS; Bladder; Irritative voiding symptoms, hematuria
Chan (1990) [14]	China; Case report	79, male	De novo MS; Kidney and prostate; Anemic, irritative voiding symptoms
John et al. (2013) [15]	USA; Case report, literature review	39, male	De novo MS; Bladder; Lower quadrant abdominal pain, nausea, vomiting, increased urinary frequency and acute renal failure
Liu et al. (2021) [16]	China; Case report (letter to editor)	24, male	Donor-derived; Kidney and ureter; Anuria
McLeod et al. (1984) [17]	USA; Case report	29, female	De novo MS; Bladder; Irritative voiding symptoms, microhematuria
Nguyen and Sayar (2018) [18]	USA; Case report, literature review	73, male	De novo MS; Prostate; Irritative voiding symptoms
Palanisamy et al. (2015) [19]	USA; Case series	Case 1: 72, male; Case 2: 77, female	Patient 1: Donor-derived; Kidney; Increased serum creatinine level 4 months post-transplant, volume overload. Patient 2: Donor-derived; Kidney; Increased serum creatinine 4 months post-transplant, recurrent urinary tract infection
Hasegeli Uner et al. (2004) [20]	Saudi Arabia; Case report	52, male	Post-transplant; Kidney; Increased serum creatinine, colic around inguinal area, occasional gross hematuria with clots
Wong et al. (2020) [21]	USA; Case report (letter to editor)	65, male	Donor-derived; Kidney; Intermittent gross hematuria
Wu et al. (2021( [22]	China; Case report	49, male	Donor-derived; Kidney; Asymptomatic, increased serum creatinine level

Our systematic literature review and the presented case underscored predominant occurrences of reported MS cases involving the urogenital tract in male patients. The reported age at diagnosis varied considerably, with the youngest case being diagnosed at age 24 and the oldest at 79 [14,16]. This is consistent with the epidemiology data reported in the fifth edition of the WHO Classification of Tumours of Haematopoietic and Lymphoid Tissues [3].

The cases identified in our literature search were distributed between de novo MS and cases arising post-transplantation or from donor-derived sources, highlighting the spectrum of origins of MS. Additionally, our findings reaffirm that the clinical presentation correlates with the specific organ involved. For instance, de novo MS involving the kidney often exhibits less distinguishable clinical symptoms, frequently presenting with tumor mass effect or hematuria. Conversely, de novo MS involving the bladder or prostate tends to manifest with irritative voiding symptoms, compression signs, and hematuria, potentially causing elevated creatinine levels and kidney dysfunction, aiding in the diagnostic process [23].

Regarding diagnostic imaging, Shallis et al. emphasized the potential role of imaging in differentiating MS from other possible pathological findings, such as abscesses or hematomas [9]. However, it is important to note that imaging is not regarded as the standard for staging MS. Our experience concurs with this, where CT and ultrasonography were the primary imaging modalities for initial screening. While these imaging modalities were useful in localizing the tumor and assessing adjacent tissue and lymph node involvement, their diagnostic value was limited compared to PET-CT scans. In line with the literature, we assert that, akin to most malignancies, there is no distinct clinical or radiographic feature that unequivocally aids in the diagnosis of MS. Therefore, the gold standard for accurate diagnosis remains immunohistochemistry, immunofluorescence, or cytogenetic analysis [15].

To validate a myeloid-origin neoplasm, several immunohistochemistry markers and cytochemical stains should be incorporated. Common markers that yield positive results in MS involving the urogenital tract are CD34, CD43, CD68, CD117, and MPO. These markers are prevalent in flow cytometric analysis for tumors exhibiting myeloid differentiation [3,15]. Based on our literature review of de novo MS involving the urogenital tract, markers that often yield positive results are CD34, CD43, CD68, CD117 and MPO. These are prevalent markers in flow cytometric analysis for tumors exhibiting myeloid differentiation [23]. Additionally, detecting cytogenetic or molecular irregularities can provide valuable insights into monitoring minimal residual disease and tailoring treatment approaches throughout a patient's therapy [15]. Detailed information is presented in Table 2.

**Table 2:** Biomarker analysis, treatment and prognosis of de novo MS of the urogenital system.

Study Citation	Biopsy and Biomarker Analysis (immunofluorescence; immunohistochemistry; cytogenetic analysis)	Treatment	Prognosis
Al-Quran et al. (2006) [10]	(Strongly positive) CD34, CD68, CD117, myeloperoxidase, lysozyme (Weakly expressed) CD45RB; 47,XY,inv(16)(p13q22)	(6 cycles) Idarubicin, cytosine arabinoside hydrochloride (high-dose)	Patient was followed up for 32 months and remains in complete remission
Acar et al. (2013) [11]	(Positive) CD34, myeloperoxidase, CD43; (Partially positive) CD68, TdT	Consolidation regimen of cytosine arabinoside and idarubicin	Not reported
Aki et al. (2002) [12]	(Positive) CD43, CD68, naphthol ASD-chloroacetate esterase, myeloperoxidase	Initially misdiagnosed and treated as round cell tumor	The patient succumbed due to sepsis during the treatment course
Chaitin et al. (1984) [13]	Author did not perform immunofluorescence, immunohistochemistry or cytogenetic analysis. Diagnosis of granulocytic sarcoma was made based pathology findings	(4 cycles) Doxorubicin vincristine, ARA-C, prednisone	Patient was followed up for 13 months and remains in complete remission
Chan et al. (1990) [14]	(Positive) chloroacetate esterase (CAE), anti-lysozyme immunoperoxidase stains	TURP	Patient succumbed after the surgery
John et al. (2013) [15]	Bladder biopsy: (positive) myeloperoxidase, CD68, CD99, CD34, CD117 Bone marrow: inv(16) involving fusion of the MYH11/CBFB genes	(“7 + 3”) Cytarabine, idarubicin infusion	Patient was followed up for 6 months and remains in complete remission
Liu et al. (2021) [16]	Ureteral mass biopsy: promyelocytic leukemia/retinoic acid receptor alpha (PML/RARα) rearrangement	Arsenic trioxide (ATO), all-trans retinoic acid (ATRA)	Patient was followed up for 4 years and remains in complete remission
McLeod et al. (1984) [17]	(Positive) myeloperoxidase	Doxorubicin, vincristine, cytosine arabinoside, prednisone	Patient was followed for 1 year and remains in complete remission
Nguyen and Sayar (2018) [18]	(Positive) MPO, CD34, CD68, with Ki-67 of 100%	(“7 + 3”) Cytarabine, idarubicin infusion; (Consolidation chemotherapy) intermediate-dose cytarabine	Patient was followed for 1 year and remains in complete remission
Palanisamy et al. (2015) [19]	Fluorescence in situ hybridization (FISH) studies suggest donor-derived myeloid sarcoma; Patient 1: (positive) CD117, CD34, MPO, myeloperoxidase; Patient 2: (positive) CD117, CD34, MPO, myeloperoxidase	Patient 1: Nephrectomy, cytarabine, daunorubicin Patient 2: Nephrectomy, patient refused systemic chemotherapy	Patient 1: Patient was followed for 8 months and remains in remission, but succumbed due to cardiovascular death; Patient 2: Patient was followed for 18 months and remains in remission, but succumbed due to cardiovascular death
Uner et al. (2004) [20]	(Positive) CD45, CD43, myeloperoxidase, CD68, CD99, Bcl-2, vimentin, S-100	Not reported	Not reported
Wong et al. (2020) [21]	Kidney biopsy: (positive) myeloperoxidase, CD117, CD33 (increased), CD34 (variably decreased), CD45 (decreased), CD13 with no expression of HLA-DR, CD4, CD14, CD15, or CD64; PML-RARα t(15;17) rearrangements	Nephrectomy	Patient succumbed due to cardiac arrest
Wu et al. (2021) [22]	(Positive) MPO, Ki-67, CD117; (Partially positive) CD34, CD68 (KP1), lysozyme, CD3, TdT Mutations of KRAS (NM_004985:exon2:c.G35A: p.G12Drs121913529), DNMT3A (NM_022552:exon15: c.1675delT:p.C559fs)	(2 cycle of DA regimen chemotherapy) Daunorubicin, cytarabine → cytarabine, etoposide	Patient was followed up for 4 years and remains in complete remission

The literature revealed instances of misdiagnosis, with reported misdiagnosis rates ranging from 25% to 75% [6,24,25]. Many undifferentiated cases were initially mistaken and treated as either urothelial or hematologic malignancies [13,26]. Our case exemplifies this challenge, where a correct diagnosis was achieved only after nephrectomy and subsequent immunohistochemistry results. Selecting an appropriate therapy necessitates an accurate diagnosis. For instance, a case described by Aki et al. initially diagnosed as transitional cell carcinoma underwent ineffective chemotherapy until the accurate diagnosis of de novo MS was established, emphasizing the pivotal role of immunohistochemistry and cytogenetic studies [12].

As of the current date, a consensus on the optimal therapy for MS remains elusive due to the lack of randomized prospective trials. Patients diagnosed with de novo MS are typically recommended systemic chemotherapy, which has shown favorable survival outcomes [23,27].

However, limited information regarding the prognosis of de novo MS involving the urogenital tract is available. Conflicting reports on the prognosis of myeloid sarcoma further underscore the need for elucidation, considering the variations in cytogenetic and molecular characteristics and the treatment approaches adopted [28,29]. Unfortunately, our patient was lost to follow-up post-nephrectomy, precluding a report on the prognosis.

## Conclusions

In conclusion, this comprehensive systematic literature review and the presented case contribute to the growing understanding of de novo myeloid sarcoma affecting the urogenital tract. The review offers valuable insights into the demographics, clinical presentation, diagnostic challenges, and treatment approaches associated with this rare condition. The multifaceted nature of MS necessitates a multidisciplinary approach, including thorough diagnostic evaluation involving immunohistochemistry, cytochemistry, and cytogenetic analysis. Standardizing terminology and refining diagnostic and treatment algorithms through future prospective studies are vital steps toward enhancing clinical management and prognosis for patients with de novo myeloid sarcoma of the urogenital tract.
